# Integrated cholangioscopy-assisted lithotripsy and a novel basket approach for managing complex cystic duct confluence stones

**DOI:** 10.1055/a-2358-1090

**Published:** 2024-07-29

**Authors:** Yasuhiro Kuraishi, Masafumi Minamisawa, Akira Nakamura

**Affiliations:** 1 Department of Gastroenterology, Shinshu University Hospital, Matsumoto, Japan

Endoscopic retrograde cholangiopancreatography (ERCP)-guided extraction is the gold standard for biliary stone management. However, cystic duct confluence (CDC) stones pose significant challenges due to their unique anatomical and stone characteristics, with a lack of consensus on the optimal therapeutic approach 1 2 . The issue is further compounded in cystic-dilated CDC spaces, in which stones are prone to escaping the retrieval catheter. Cholangioscopy-assisted lithotripsy is a promising technique that facilitates complex stone management through direct visual stone fragmentation 3 . Featuring a helical 8-wire design with a narrower interwire space at its tip and rotational capability, the novel basket (RASEN2; KANEKA Medix, Tokyo, Japan) has shown superior stone clearance versus conventional baskets in an experimental ex vivo setting 4 . We describe an integrated approach using cholangioscopy-assisted lithotripsy and RASEN2 for CDC stone removal.

A 31-year-old man was diagnosed at a prior hospital with recurrent cholecystitis and cholangitis due to a large stone impacted at the CDC ( Fig. 1 ). Initial attempts of ERCP-guided extraction were thwarted by the stone’s impaction and anatomical complexity, rendering mechanical lithotripsy ineffective. Temporary biliary stenting and sphincterotomy were performed. The extensive adhesions accompanied by persistent peri-gallbladder inflammation made surgical intervention infeasible. We performed cholangioscopy-assisted lithotripsy following patient referral to our institution ( Fig. 2 , Video 1 ).

**Fig. 1:**
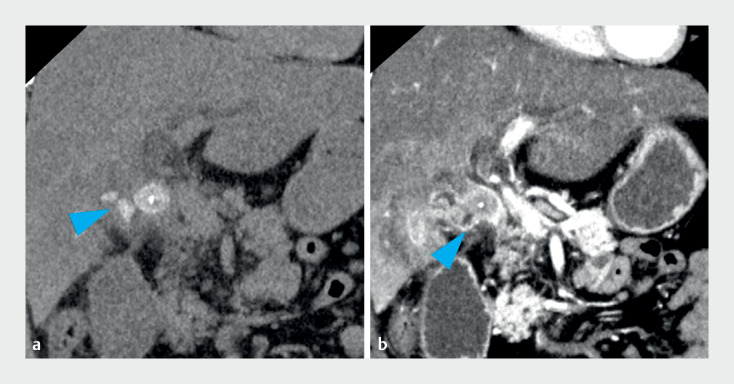
Computed tomography images. **a,b** A large stone (arrowheads) was impacted at the cystic duct confluence, leading to cholestasis in both the bile duct and cystic duct.

**Fig. 2:**
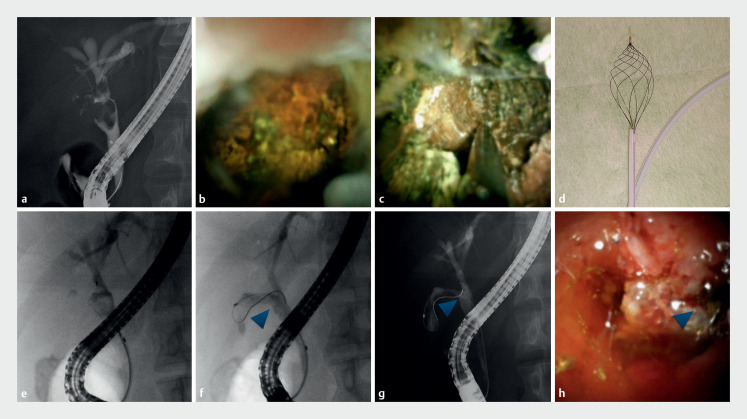
Cholangioscopy-assisted lithotripsy procedure. **a** Cholangiography revealed a significant obstruction at the cystic duct confluence (CDC) caused by a large stone. The dilation around the CDC stone formed a pocket that complicated extraction. **b** Cholangioscopy showed the CDC stone fully occupying the ductal lumen. **c** Electrohydraulic lithotripsy performed under saline irrigation successfully fragmented the CDC stone. **d** the RASEN2 basket (KANEKA Medix, Tokyo, Japan), featuring a novel helical 8-wire design with a smaller interwire space at the proximal tip and rotation function, was employed for the stone extraction. **e** The rotation and enhanced expansion ability of RASEN2 enabled effective stone extraction from the dilated CDC, ensuring secure contact with the duct wall and complete stone capture. **f** The stones in the cystic duct could also be removed by RASEN2. **g** Post-extraction cholangiography showed the absence of residual stones at the CDC. **h** Cholangioscopic assessment confirmed the complete clearance of stones from both the bile duct and cystic duct. Arrowheads indicate the junction point of the cystic duct.

Synergistic application of cholangioscopy-assisted lithotripsy and the RASEN2 basket (KANEKA Medix, Tokyo, Japan) was an effective therapeutic approach for cystic duct confluence stones.Video 1

Under cholangioscopy visualization (SpyGlass DS; Boston Scientific, Marlborough, Massachusetts, USA), a large CDC stone occupying the lumen was identified and then successfully fragmented using electrohydraulic lithotripsy. Attempts to capture the fragmented stone with conventional retrieval basket and balloon catheters failed due to the enlarged CDC. However, switching to the RASEN2 basket facilitated fragment removal by navigating the complexities of the dilated lumen, which ultimately led to complete stone clearance as confirmed by cholangioscopic and cholangiographic assessments. Post-procedure, the patient experienced no recurrences of cholecystitis or cholangitis.

The synergistic application of cholangioscopy-assisted lithotripsy and RASEN2 appears to be an effective approach for challenging CDC stones.

Endoscopy_UCTN_Code_TTT_1AR_2AH

## Notes

Endoscopy E-Videos https://eref.thieme.de/e-videos
*E-Videos* is an open access online section of the journal *Endoscopy* , reporting on interesting cases and new techniques in gastroenterological endoscopy. All papers include a high-quality video and are published with a Creative Commons CC-BY license. Endoscopy E-Videos qualify for HINARI discounts and waivers and eligibility is automatically checked during the submission process. We grant 100% waivers to articles whose corresponding authors are based in Group A countries and 50% waivers to those who are based in Group B countries as classified by Research4Life (see: https://www.research4life.org/access/eligibility/ ). This section has its own submission website at https://mc.manuscriptcentral.com/e-videos .

## References

[1] Marcelino LP Thofehrn S Eyff TF Factors predictive of the successful treatment of choledocholithiasis Surg Endosc 2022 36 1838 1846 10.1007/s00464-021-08463-5 33825014

[2] Tringali A Costa D Fugazza A Endoscopic management of difficult common bile duct stones: where are we now? A comprehensive review World J Gastroenterol 2021 27 7597 7611 10.3748/wjg.v27.i44.7597 34908801 PMC8641054

[3] Bhandari S Bathini R Sharma A Usefulness of single-operator cholangioscopy-guided laser lithotripsy in patients with Mirizzi syndrome and cystic duct stones: experience at a tertiary care center Gastrointest Endosc 2016 84 56 61 26764195 10.1016/j.gie.2015.12.025

[4] Inoue T Ibusuki M Kitano R Comparison of the removal ability of basket catheters for small bile duct stones impacted in the corner pocket of the lower bile duct Endoscopy 2022 54 987 992 10.1055/a-1769-0514 35144287

